# Music therapy versus treatment as usual for refugees diagnosed with posttraumatic stress disorder (PTSD): study protocol for a randomized controlled trial

**DOI:** 10.1186/s13063-018-2662-z

**Published:** 2018-05-30

**Authors:** Bolette Daniels Beck, Steen Teis Lund, Ulf Søgaard, Erik Simonsen, Thomas Christian Tellier, Torben Oluf Cordtz, Gunnar Hellmund Laier, Torben Moe

**Affiliations:** 1 Department of Communication and Psychology, Aalborg University, Aalborg, Denmark; 2 Clinic for Traumatized Refugees, Køge, Region Zealand Denmark; 3 Department of Specialized Functions, Psychiatry, Køge, Region Zealand Denmark; 4 Institute for Clinical Medicine, SUND, Copenhagen University, København, Denmark; 5 Research Unit in Psychiatry, Slagelse, Region Zealand Denmark; 6 PFI (Production, Research, Innovation), Sorø, Region Zealand Denmark

**Keywords:** Music and imagery, Refugees, Randomized clinical trial, PTSD, Trauma, Music therapy, Non-inferiority, Oxytocin

## Abstract

**Background:**

Meta-analyses of studies on psychological treatment of refugees describe highly varying outcomes, and research on multi-facetted and personalized treatment of refugees with post-traumatic stress disorder (PTSD) is needed. Music therapy has been found to affect arousal regulation and emotional processing, and a pilot study on the music therapy method Trauma-focused Music and Imagery (TMI) with traumatized refugees resulted in significant changes of trauma symptoms, well-being and sleep quality. The aim of the trial is to test the efficacy of TMI compared to verbal psychotherapy.

**Methods:**

A randomized controlled study with a non-inferiority design is carried out in three locations of a regional outpatient psychiatric clinic for refugees. Seventy Arabic-, English- or Danish-speaking adult refugees (aged 18–67 years) diagnosed with PTSD are randomized to 16 sessions of either music therapy or verbal therapy (standard treatment). All participants are offered medical treatment, psychoeducation by nurses, physiotherapy or body therapy and social counseling as needed. Outcome measures are performed at baseline, post therapy and at 6 months’ follow-up. A blind assessor measures outcomes post treatment and at follow-up. Questionnaires measuring trauma symptoms (HTQ), quality of life (WHO-5), dissociative symptoms (SDQ-20, DSS-20) and adult attachment (RAAS) are applied, as well as physiological measures (salivary oxytocin, beta-endorphin and substance P) and participant evaluation of each session.

**Discussion:**

The effect of music therapy can be explained by theories on affect regulation and social engagement, and the impact of music on brain regions affected by PTSD. The study will shed light on the role of therapy for the attainment of a safe attachment style, which recently has been shown to be impaired in traumatized refugees. The inclusion of music and imagery in the treatment of traumatized refugees hopefully will inform the choice of treatment method and expand the possibilities for improving refugee health and integration.

**Trial registration:**

ClinicalTrials.gov ID number NCT03574228, registered retrospectively on 28 June 2016.

**Electronic supplementary material:**

The online version of this article (10.1186/s13063-018-2662-z) contains supplementary material, which is available to authorized users.

## Background

Currently, there are more than 21 million internationally displaced refugees in the world, 3.5 million of them living in Europe [1]. The mental health problems and psychosocial strain in refugees resettled in Western countries has been suggested to relate to traumatic experiences and stress while living under war, persecution and other life-threatening circumstances, danger and challenges during flight, as well as post-migration experiences such as insecure waiting periods during asylum and family reunion procedures, poverty, lack of social support, acculturation difficulties and discrimination [2]. Reviews of studies on refugee health document a large variation in health status related to country of origin, country of resettlement and the methodological quality of the studies. In a systematic review of 20 surveys including 6743 adult refugees from seven countries a 9% prevalence of PTSD (99% CI 8–10%) was found, which is about ten times the rates in the age-matched general populations in the same countries [3]. In a systematic review of 29 studies including 16.010 war-affected refugees, significant between-study heterogeneity in prevalence rates of post-traumatic stress disorder (PTSD) (4–86%), unspecified anxiety disorder (20–88%) and depression (range 2–80%) was identified, although prevalence estimates typically were in the range of 20% and above [4]. All three disorders were associated with greater exposure to pre-migration trauma and post-migration stress, while depression was particularly associated with poor socio-economic status. In a study of 142 newly arrived asylum seekers in Denmark 34% had symptoms corresponding with the PTSD diagnosis [5].

In *International Classification of Diseases* (ICD-10) criteria for PTSD are the exposure to an exceptionally threatening event of catastrophic nature and demonstrating the symptom triad (1) intrusive trauma-related imagery or nightmares, (2) avoidance of situations that reminds them of the trauma and (3) either (a) partial amnesia of the trauma or (b) prolonged hypervigilance that causes irritability or frequent outburst of anger, concentration problems, sleeping problems and/or exaggerated startle response (F43.1, World Health Organization (WHO) ICD-10). In addition, PTSD influences cognitive abilities such as memory and learning, and often causes social withdrawal, all of which affect quality of life in a profoundly negative way, making it harder for the affected individuals to achieve successful integration and self-perseverance, including the adherence to study and work. In the upcoming ICD-11 the diagnosis Complex PTSD will be included, a diagnosis for additional symptoms related to longitudinal and/or severe traumatic exposure characterized by disturbances in emotional regulation and relational capacities, dissociation, somatic distress and alterations in belief systems [6]. In the draft for the upcoming ICD-11 the following symptoms are listed: (1) severe and pervasive problems in affect regulation; (2) persistent beliefs about oneself as diminished, defeated or worthless, accompanied by deep and pervasive feelings of shame, guilt or failure related to the traumatic event; and (3) persistent difficulties in sustaining relationships and in feeling close to others (ICD-11, draft version). In line with that, insecure attachment recently has been shown to be common in refugees [7, 8]. Furthermore, refugees from non-Western countries show high levels of unexplained somatic symptoms that potentially can be explained by traumatization, the results of torture and that also might be a culturally accepted way to express psychological pain taking into consideration the stigmatization of psychiatric care [9]. The increased number of refugees with severe mental health problems necessitates the development of effective treatment modalities.

### Research on refugee treatment

Standardized short-term treatment has been shown to be effective in the treatment of single-trauma PTSD without significant comorbidity [10, 11]. However, simple PTSD is not typical in traumatized refugees, where complex trauma and comorbidity is common. The recommendation from leading trauma researchers in complex PTSD is a treatment based in a cross-disciplinary setup with a phased psychotherapeutic component of a duration of several years [6].

Until today the evaluation of psychological treatment modalities of traumatized refugees has mostly focused on individual cognitive behavioral therapy (CBT) and narrative exposure therapy (NET), with some studies in eye movement desensitization reprocessing (EMDR), combined methods/interdisciplinary treatment and group treatment. Compared to other patient groups, the evaluation of psychotherapeutic treatment of refugees can be difficult, randomized controlled trials are few and sample sizes are generally small. There are many possible explanations for this. Many refugees that have experienced traumatic events, such as persecution, are reluctant to trust authorities, including healthcare personal. The complexity of trauma symptoms, acculturation difficulties and the influence of the translator upon the psychotherapeutic relationship also have to been taken into account.

In a Cochrane review evaluating nine studies on CBT and NET with torture survivors, no effect was found immediately after treatment. However, at 6 months’ follow-up, four out of nine studies showed a medium effect on trauma symptoms [12]. Slobodin and de Jong [13] performed a meta-analysis of studies with quantitative pre-post intervention measurement of trauma symptoms, including refugees and asylum seekers. They found positive effects on PTSD symptoms after treatment with CBT and NET in certain refugee populations. Other intervention studies, i.e., EMDR, psychodynamic interventions, family interventions, group interventions, pharmacological treatment and combined methods/interdisciplinary treatment, were limited by methodological considerations, such as lack of randomization, absence of control group and small samples. Similar conclusions were found in previous academic literature reviews [14–17]. Palic and Elklit [17] reviewed 25 refugee studies and found that the effect of different approaches varied from very small to medium effect sizes. Among the reviewed studies a few demonstrated very large effect sizes on PTSD symptoms after trauma-focused phased CBT with a body-oriented and culturally sensitive approach. In these approaches CBT was combined with guided imagery with culturally specific images such as a flowering lotus [18–20] or cognitive restructuring was combined with progressive relaxation, affect regulation skills and guided imagery, such as imagination of a safe place [21]. These approaches seem to resemble the music therapy method applied in the present clinical trial.

Lambert and Alhassoon [22] aggregated the effect sizes for trauma symptoms and depression in 13 randomized controlled trials of different psychotherapeutic interventions for traumatized adult refugees. They found a large aggregate effect size for PTSD that was independent of type of outcome measure (Hedges’ *g* .91, *p* .001, 95% CI [.56, 1.52]. Depression was assessed in nine studies, and here the effect size was also large (Hedges’ *g* .63, *p* .001, 95% CI [.35, .92]. Higher number of sessions (3–12) predicted the magnitude of PTSD change significantly. Translated and untranslated sessions were compared and there was no visible effect of the translation process upon the clinical outcome of the studies.

Because of the complex situation of traumatized refugees and the fact that the majority of refugees remain chronically traumatized, several of the reviews maintain, that other measures than the level of trauma symptoms have to be recognized when evaluating the effect of specific therapies in this patient group. These include the evaluation of long-term treatment effects, social functioning, improvements in the capacity for maintaining meaningful relationships, and a positive experience of identity and meaning [13, 17]. Recently, the investigation of attachment-based treatment strategies, and the inclusion of attachment style as outcome measure has been recommended [7, 8]. Furthermore, treatments that could prove effective in preventing relapse should be further investigated. These include methods that address affect dysregulation and coping strategies targeted to the ongoing insecurity and uncertainty typical for the life situation of refugees [16, 17]. Personalized treatment is currently being introduced in many psychiatric clinics. In line with that Slobodin and de Jong [13] argued that it is necessary to devise the treatment of refugees individually from a spectrum of possibilities.

#### Physiological measures and PTSD

Levels of stress hormones, such as cortisol have been widely used to measure the stress response in PTSD patients. However, several studies have failed to confirm the expectation that cortisol is elevated in PTSD. On the contrary, patients who suffer from PTSD generally have normal levels of cortisol, and often their levels are even lower than the values of healthy participants [23, 24]. It has been supposed that the HPA axis suppresses the cortisol response in PTSD (or vice versa), making this hormone difficult to use as an outcome measure in trials [25].

Oxytocin is a neuropeptide that is involved in the regulation of fear. It also enhances trust and prosocial behavior, and it has been associated with stress reduction, wound healing, attachment, calmness and rest [26]. It has even been suggested that oxytocin could be administered intranasally in the treatment of PTSD, especially it has been considered for early prevention, and as a treatment to increase social responsiveness [27, 28].

In a meta-review of 400 studies investigating the effect of music on brain chemistry, it was found that music contributes to the production of peptides such as oxytocin, vasopressin and dopamine that add to the creation of social bonding, and endogenous opioids that contribute to the maintenance of steady social relationships [29]. A number of music intervention studies have demonstrated increased peripheral oxytocin levels after post-operative music listening [30], singing lessons or improvised singing [31–33] and choir singing [34]. Beta-endorphin is also associated with the stress response and low levels have been implicated in PTSD [35]. Beta-endorphin was lowered in healthy undergraduates after music and imagery [36] and in patients with coronary heart disease after music listening [37].

### Research on music therapy with refugees

For decades, music therapy as a clinical psychotherapy model has been applied to a broad spectrum of populations in the health system [38]. Recent Cochrane reviews of music therapy treatment have demonstrated moderate-quality evidence on reduction of depression in people with dementia [39] improvement of walking in people with stroke [40] and increase of social communication skills in children with autism [41]. In a Cochrane review of people with schizophrenia, it was concluded that music therapy as an addition to standard care improved global state, mental state (including negative symptoms) and social functioning [42]. A selective review of music therapy studies with PTSD patients concluded that individual and group interventions seem to reduce core PTSD symptoms and depression and increase social function, hope and resilience in both adults and children [43].

A randomized controlled study including adult psychiatric patients with persistent PTSD, who had been unable to benefit from CBT, showed a significant decrease of all dimensions of PTSD symptoms after group music improvisation compared to a waitlist control [44]. Studies in group music therapy with children and adolescents showed some effect on PTSD symptoms after four sessions of songwriting compared to games in nine psychiatric patients aged 9–17 years with histories of sexual abuse [45]. Two Australian school studies demonstrated beneficial effects of music therapy: In 31 newly arrived refugees, a decrease in hyperactivity, aggressive behavior, depression, anxiety and somatization were found in periods with music therapy compared to periods without music therapy [46]. In a randomized study, reduction of depression, hopelessness and anxiety after group music therapy compared to art classes was demonstrated (*n* = 18) [47, 48].

Jespersen and Vuust [49] found significant improvement of sleep quality in a randomized controlled trial of adult refugees with undiagnosed PTSD (*n* = 23). All participants slept on a special pillow with loudspeakers; one group listened to calming music 30 min before falling asleep, the other group had no music. In another sleep study, music-guided relaxation resulted in decreased depression and increased sleep quality compared to relaxation with no music in 13 veterans [50]. Akhtar [51], adapting a quasi-experimental design, found beneficial changes in depression and anxiety in a small group of Pakistani traumatized refugees after music listening to live improvised music (in combination with treatment as usual). Likewise, Alanne [52] found improvements in depression and quality of life in follow-up case studies of three torture survivors. Using a factor analysis of 106 sessions he demonstrated improvement in the ability of the refugees to contain, process and contain emotions and traumatic events from before to after music listening [52].

#### Guided Imagery and Music (GIM)

The Bonny method of GIM is facilitating music-evoked imagery in an altered state of consciousness as an in-depth psychotherapeutic method. The original method applies 30–45 min of listening to carefully selected movements of classical Western music [53, 54]. Adaptations of the method to trauma survivors have been promising. In a naturalistic study of 102 women suffering from Complex PTSD, 50 h of GIM resulted in significant improvement of PTSD symptoms, decreased symptoms of dissociation and better “sense of coherence” with large effect sizes compared to PITT (imagery-based therapy without music) [55]. In psychiatric patients, GIM in group treatment conveyed restitution and increased affect regulation [56, 57] and reduced trauma symptoms [58]. In a psychiatric group treatment program, including patients with refugee background, the outcome measures showed better outcomes of GIM in trauma victims than in patients without trauma history [59]. Two pilot studies with trauma survivors demonstrated large effect sizes of GIM interventions. In one study, ten women with histories of sexual/physical childhood abuse who suffered from complex or single PTSD participated in 12 sessions of trauma-focused group GIM. All of the participants achieved significant symptom relief of PTSD symptoms, dissociation, anxiety and depression [60]. In another study, the effect of ten sessions of individual resource-oriented GIM was assessed in female veterans, who had been subjected to sexual abuse. Focus group interview analysis found that music therapy succeeded in helping the victims cope with their PTSD symptoms, regulate their emotions, decrease arousal, express repressed emotions and connect better with others. Creative processing (drawing the imagery) provided increased creative expression and a way to continue processing between sessions [61].

### Pilot study

The background for the present study is a completed feasibility study in the form of a pilot project concerning treatment of traumatized refugees with PTSD in Region Zealand, Denmark [62]. In the 1-year-long project, an adaptation for trauma treatment of GIM called Trauma-focused Music and Imagery (TMI) including the central elements music listening and imagery was applied to 16 adult participants. Participants were ten men and six women of different origin (Syria, Afghanistan, Iraq, Iran) with a mean age of 40 years. All participants completed the 16 sessions, but weekly sessions were not always possible due to cancellations; therefore, length of treatment was on average 26 weeks. The single group pre-test/post-test study showed significant positive changes with large effect sizes (0.81–1.17) on PTSD symptoms, well-being, sleep quality and social function. Symptom load measured with the Harvard Trauma Questionnaire, showed both significant change (*p* < 0.002) and large effect size (1.15). Three participants scored under cutoff for PTSD after treatment. Patient satisfaction was measured with a 7-point smiley scale from “very dissatisfied” to “very satisfied.” The average score was 5.5 with a slightly higher satisfaction towards the end of treatment. According to post-treatment interviews the patients experienced improvement as a result of treatment, and they experienced music as important for coping and emotional regulation. Furthermore, the music influenced the restoration of trust and hope, both of which are known to be involved in the achievement of secure attachment. The music repertoire used included Arabic and Afghan pieces, as 25% of the participants needed familiar music to work with their inner images. All participants used the music method at home for self-care, relaxation, affect regulation, release of pain symptoms, and positive focusing.

In order to assess the positive outcome of the pilot study in a larger study, the randomized clinical trial that is presented here was established.

### Rationale for the randomized trial

The increased number of traumatized refugees and their generally problematic situation cause pressure on the treatment systems in the host countries. Accordingly, effective treatment options are needed in order to help the refugees to attain positive integration and a better quality of life. To our knowledge, no former randomized studies on music therapy with adult refugees exist, and no known refugee studies include physiological measures. The study hereby contributes to the investigation of music therapy and the GIM method as an effective treatment modality. Verbal psychotherapy carried out by psychologists is chosen as comparator because it is the standard treatment modality in the field, and because the effect of music therapy versus verbal therapy can be investigated. Due to the limited evidence and knowledge about the efficacy of existing treatment programs on key symptoms related to the complex traumatizing of refugees, including unsafe attachment patterns and dissociation, the study attempts to add to the knowledge of the efficacy of both.

## Methods

### Aim

The aim of the study is to create increased treatment modalities for refugees with PTSD, and to provide new knowledge about the efficacy and non-inferiority of music therapy compared to standard verbal psychotherapy as primary psychotherapeutic methods in refugee treatment. The study also seeks to point out possible parameters that can aid as a help in the clinical referral procedure and guide the choice of one or the other treatment modality.

Furthermore, the study aims to investigate the change of symptoms on several parameters that earlier have been tested in a pilot study as well as parameters that have not been tested before (dissociation and attachment). As an extension of the study, molecular data (salivary oxytocin, beta-endorphin and substance P levels) are included as possible outcome measures.

### Research questions

The following research questions are posed:

1. Will music therapy (TMI) be as effective as standard treatment regarding the decrease of TSD symptoms (primary outcome) from pre to post treatment and from pre-treatment to 6 months’ follow-up?
2. Will music therapy (TMI) be as effective as standard treatment regarding the decrease of dissociation and the improvement of attachment style and well-being (secondary outcomes) pre to post treatment and from pre-treatment to 6 months’ follow-up?
3. Does any variable from the patient data at baseline (i.e., gender, age) specifically correlate with good outcomes of music therapy on trauma symptoms compared to standard treatment?
4. Concerning patient satisfaction with therapy sessions, is there a difference between music therapy and verbal therapy, and is there any development in the assessment of the sessions during the therapy course?
5. Will basic levels of salivary oxytocin, beta-endorphin and substance P be changed by the treatment, are any changes stable at follow-up, and are there differences between the two groups?
6. Will the levels of salivary oxytocin, beta-endorphin and substance P be affected by one session, and are there a difference between the groups, and between a session at the beginning and in the end of treatment?

#### Hypotheses

The hypotheses of the study are that music therapy (TMI) will not be less effective than verbal psychotherapy according to the principle of clinical equipoise, and that we will see a decrease in PTSD symptoms and dissociation as well as an increase of well-being and improvement of safe attachment style after both music therapy and standard treatment. Furthermore, we hypothesize that patient evaluation will be equally positive with regard to both treatment conditions. According to salivary hormones the hypotheses are, that music therapy will be no less effective than verbal therapy regarding increase of basic and session levels of oxytocin and decrease of basic and session levels of beta-endorphin and substance P.

### Trial design

**Fig. 1:**
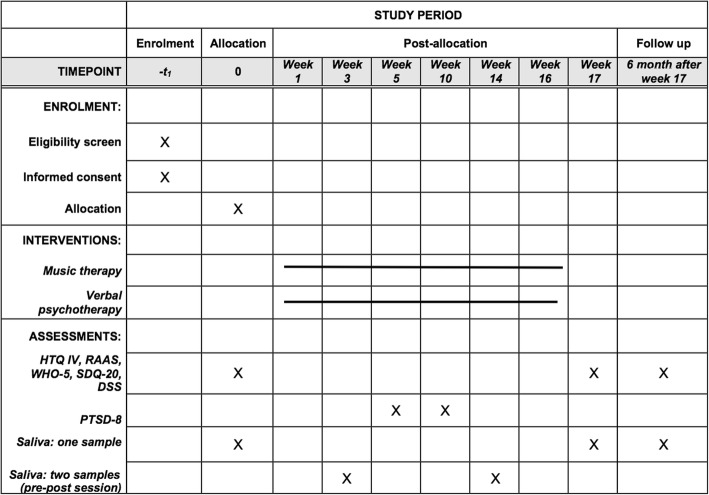
Schedule of enrollment, interventions, and assessments (Standard Protocol Items: Recommendations for Interventional Trials (SPIRIT) flow chart)

The research design is a randomized clinical trial with a parallel-group design including two intervention groups: music therapy (Trauma-focused Music and Imagery (TMI)) and standard psychological treatment. We use a non-inferiority framework, where we test whether or not music therapy is inferior to standard treatment. We intend to allocate 70 adult refugees diagnosed with PTSD. Repeated measures take place at baseline, post therapy and at 6 months’ follow-up. A short form of the primary outcome measure is also collected twice during the treatment period (session 5 and 10). Session evaluation from clients is collected after each session. Saliva samples are collected at baseline, post therapy and follow-up, and also pre and post sessions 4 and 14 (see the Standard Protocol Items: Recommendations for Interventional Trials (SPIRIT) flow chart; Fig. 1). All dimensions of the study protocol have been described adhering to the SPIRIT Checklist (Additional file 1). We also adhere to the revised Consolidated Standards of Reporting Trials (CONSORT) guidelines [63].

### Setting

The study is carried out in the outpatient Trauma Clinic for Refugees under the Department of Special Functions in Psychiatry, Region Zealand, Denmark. The clinic offers treatment in three units placed at different locations in the area a multidisciplinary treatment including a combination of medication, psychotherapeutic treatment, social counseling, health advice given by nurses, body therapy and music therapy. Translators are used as needed. Around 250–300 patients are referred per year, most from general practitioners and some from the Centers of community psychiatry. Patients must have a residence permit to access the clinic. The average treatment time is 4–6 months. When a patient is referred to the clinic without a PTSD diagnosis, the physician and psychologist assess the patient. As part of the visitation and screening procedure in the clinic, the physician and the treatment team estimate a mentalization level (small, medium or high) for the patient, based on the actual psychosocial resources and capacity for introspection, reflection, affect regulation and empathy [64, 65].

The trial covers all three locations of the clinic. A list of the places can be found in the Appendix. Four psychologists and four music therapists are responsible for the treatment.

### Participants

The participants are adult refugees (age 18–67 years) accepted for treatment by the Clinic of Traumatized Refugees, i.e., by way of having a psychic trauma related to war, persecution, torture, etc. Diagnoses for inclusion are: PTSD (ICD-10: F43.1 or DSM-IV-TR: 309.81), Enduring personality change after catastrophic experience (ICD-10: F62.0), or Complex PTSD/DESNOS (Disorders of Extreme Distress Not Otherwise Specified) (DSM-V). Comorbidity, such as depression, anxiety disorders, non-psychotic depression, episodic psychotic symptoms related to (Complex)PTSD, somatoform disorders or personality disorders, are accepted in the study.

Medication for PTSD and comorbid illness as well as for somatic diseases are accepted. The participants can be refugees or family united with refugees from all countries. Included are Arabic-, English- or Danish-speaking participants. These criteria are chosen to secure access to self-report questionnaires in a language understood by the participants, thereby avoiding misinterpretation of the questions. Included in the study are participants with a medium to high mentalization level (as estimated in the clinic after the visitation procedures). All participants receive verbal and written information in their native language and are included after giving informed consent to participate in the study.

#### Exclusion criteria

Exclusion criteria are severe psychotic disorders, defined as psychotic disorders in the domain of F2 and F3 in ICD-10. No active abuse was accepted (ICD-10 F10.24–F10.26), and persons requiring hospitalization are not included. Patients with suicidal risk at referral are not included in the study.

#### Eligibility criteria for psychotherapists

Psychologists carrying out standard treatment in the study are employed as psychologist in the Clinic for Traumatized Refugees; the music therapists have to have a master’s degree in music therapy and/or be a certified GIM therapist, and be employed in the clinic.

### Recruitment

The visitation procedure of the Trauma Clinic for Refugees includes: consultation with a physician regarding diagnostics, medication and motivation for treatment, consultation with a nurse to obtain information about health, consultation with a psychologist to obtain an overall record of trauma history and background, coping strategies and attachment style. Assessment of biopsychosocial competencies and mentalization level are carried out by the cross-disciplinary team at conferences.

#### Procedure of obtaining informed consent

Following the regulations of the regional Ethical Scientific Committee, patients eligible to participate in the trial are invited to an information meeting with one of the music therapists or the senior staff specialist, where they are informed orally as well as provided with a written description of the study and of their rights as participants that is available in Arabic, Danish and English. A translator is present to translate if necessary, and the patient is allowed to bring a companion. The meeting takes place in calm surroundings, and there is time set aside for questions. The patient is given information about the purpose of the project, the content, pros and cons for participants, and their rights as a participant in a scientific health study. The principle of randomization is explained, and both treatment options are described, so that the participants only give informed consent when understanding their options. The patient is given information about their right to withdraw from the study at any time without missing out on options for current and future treatment. The patient is informed that they will be informed if important health issues are revealed during the study, unless they do not want to receive this information, and that at anytime they can ask for sight of their personal data and withdraw such data from the project. The patient gives permission for the data to be analyzed and published anonymously. The patient is given one week to consider before giving informed consent. If the patient gives consent directly after the information, they sign the informed consent on the spot. If the patient needs time to consider their participation a telephone call is scheduled a week later, and a new meeting is set up for the signing procedure.

A participant is included in the study when we have obtained informed content. The leader of the study and the patient each have a copy of the informed consent contract (see Additional file 2).

#### Feasibility

The pilot study leading up to the current study [62] had a high retention rate (no dropouts), the patients showed a high degree of compliance. Filling in outcome self-report measures was feasible with the help of the translators. Recruitment took place in cooperation with the physician in only one clinic. In the current study, three locations with 250–300 patients per year are included, and there are 1.5–2 years to recruit 70 participants. The potential participants are screened for eligibility already during the clinical visitation process. We are aware that music therapy is a new treatment option that can seem difficult to understand by some patients, that the randomization process has to be explained carefully and that participation in a research study can be a stress factor for traumatized patients. We assume that the feasibility of performing a RCT within the chosen framework will be reasonable.

## Interventions

The refugee clinic carries out trauma treatment in a multidisciplinary clinical setting. The current study compares only the psychotherapeutic part of treatment, but for ethical reasons all other services (social counseling, body therapy, health advice) in the clinic are kept open for all participants as needed, and the extent of other services will be monitored throughout the study. All participants who receive medication as part of the treatment in the experimental group as well as the standard treatment group are monitored continuously by the physicians during the whole course of psychotherapy. Differences between groups are analyzed as possible confounders related to outcome.

### Dose

A significant correlation between dose and response has been found for music therapy treatment with serious mental disorders, with small effect sizes after three to ten sessions and large effects after 16 to 51 sessions [66]. A review of quantitative studies of GIM concluded, that at least ten sessions should be provided for clinical populations [67]. In our pilot study, 16 sessions provided significant changes with large effect sizes on all the outcome measures. This number of sessions also fits with the psychiatric clinic’s standard length of treatment. We chose to repeat the 16 sessions as dose for both treatment arms in the present study.

The patients participate in weekly sessions, lasting 60 min. If any cancellations occur the treatment period is prolonged until all 16 sessions have been received. Should the participant encounter any given events that would lead to further traumatizing by way of circumstances outside therapy, treatment can be prolonged to as many as 20 sessions, after consultation with the treatment team and the physician. Hence, a margin of 25% is accepted (from 12 to 20 sessions). In both music therapy and the comparator treatment a phase-oriented treatment according to Herman [68] is carried out, including a stabilizing phase, a trauma-exposure phase and a reorientation phase (if possible). The development of a safe therapeutic relationship and verbal processing are components in both psychological standard treatment and trauma-focused GIM. Thereby, the professional adaptation of music as a therapeutic medium, including the way that music influences the therapeutic relationship, is the independent variable in the trial.

### Trauma-focused Music and Imagery (TMI)

TMI is an adaptation of GIM, a music therapy method initiated by the American music therapist Helen Bonny in the 1970s [53, 69] using music listening of selections of classical music in an altered state of consciousness as a medium for therapeutic change. During the listening experience, a non-directive verbal dialog between patient and therapist is carried out supporting the deepening and integration of the ongoing stream of imagery, emotions and sensations evoked by the music. GIM has served as primary psychotherapeutic treatment in a range of clinical settings and has been modified and adapted to several clinical populations [70]. A continuum of practices exists, from the full Bonny method (2-h sessions with challenging music and verbal dialogue) to short GIM (1-h sessions, shorter music selection); music and imagery being a supportive short-form that often only includes one or a few short music pieces and no verbal interaction during the music listening part.

A GIM therapist holds a master’s degree in music therapy or other relevant subject followed by a minimum of 3 years of supervised training in the GIM method licensed by an international training institute (see www.music-and-imagery.eu).. The method has been adapted to refugee trauma treatment by the authors Beck, Moe and Meyer [62].

A TMI session includes:

- A verbal conversation used to check in and talk about actual issues
- Agreement of a focus for the music listening
- Finding a music piece (patient chosen or therapist chosen; if the therapist chooses the piece a small excerpt is played to assess the match of music with the patient). Music from several genres are optional: classical, film, meditative music, or music corresponding to the patient’s cultural background
- Music listening; the patient is sitting on a chair or lying on a couch, eyes open or closed as preferred
- A short induction; for example, mindful focus on breathing, guided relaxation or focus on an inner image
- Music listening (2–10 min in the beginning, up to 20 min in the end of therapy). The therapist can talk during the music if helpful
- The therapist guides the patient back to the present
- Participants are invited to draw a picture of the imagery
- Verbal communication about the drawing and the experience with the focus on integration of important imagery, acknowledgement and meaningfulness in the therapeutic process
- Homework assignment as needed (including using music at home)

Treatment phases (the session numbers are indicative as a patient can stay in phase 1 for the whole time or go back and forth between phases):

#### Phase 1. Stabilization phase (sessions 1–5)

The participants are provided with a CD containing seven pieces of music for listening to at home (see Appendix 2). The preferred piece(s) is(are) used in the first sessions. The patient’s relationship with music is explored. Psychoeducative elements are introduced (such as understanding of PTSD symptoms and the autonomic nervous system in trauma). Music accompanied breathing [70] is offered as help to deepen the breathing (abdominal breathing) and to regulate arousal. Positive inner imagery such as “the safe place” and positive memories of close relatives or events before the war are presented as central to increase biopsychological resources and safety. Music as a safe ground and as a way to detach from pain and negative feelings is in the focus. The therapeutic alliance develops through shared experience with music. The music in the stabilization phase is characterized by a high degree of predictability concerning the musical parameters (i.e., a stable, slow tempo, only gradual changes in volume, rhythm, sound, register and harmony), simple dynamics (such as ABA) and use of repetition in melody and chords. The participant is asked to work with the music at home between sessions.

#### Phase 2. Emotional containment (sessions 6–8)

Contact with different kinds of emotions in the music is explored. Both positive and difficult emotions can be experienced, while listening to music, and the ability to contain contrasting emotions, ambivalence and different aspects of emotions are important parts of the investigation process. The primary focus is on giving the patients an opportunity to both explore and be able to stay with difficult emotions with the aid of music, as well as a possibility to develop new coping skills that will allow the patients to change their emotions through their interaction with the music. If the patient is stable enough and feels safe, music with more depth and dynamics can be introduced.

#### Phase 3. Trauma exposure and grieving (sessions 9–14)

A process of exposure during music listening is carried out when the patient has achieved sufficient stabilization. The narrative of traumatic events can be accompanied by, and supported by, music, or music and imagery can be used for exploration of traumatic episodes. Trauma imagery can also emerge during music listening without a fixed focus, and can be processed with the support of the therapist and the music. The music serves as a holding structure that match the emotions and states of trauma processing, or the music can be used to regulate arousal during exposure. All sessions are carried out with the focus on step-by-step work and safety; for instance, can music pieces connected to safety imagery or positive resources alternate with pieces of music accompanying trauma memory. Traumatic episodes can be renegotiated during music listening, meaning that the patient finds alternative solutions in imagery to a stuck situation in the past, and/or that incomplete defense actions (fight and flight) can be carried out in the imagination. Grieving and loss are common themes that are explored in the music and imagery experiences, and can be assessed by encouraging the patient to engage in imaginary dialog with lost relatives. Anger management can be included as a therapeutic focus.

If a patient is overwhelmed by the music and imagery experience, or suffers intruding flash-backs, the music is immediately turned down or changed. When possible, music from the patient’s own culture is evaluated by the interpreter before use to ensure that the lyrics and the traditional use of the piece is appropriate for the session.

#### Phase 4. Reorientation (sessions 14–16)

In phase 4 the patients are encouraged to develop their social network and engagement in activities in their community if they are ready for it. Music and imagery can help the patient to be reminded of their dreams, and to keep them in focus as a beacon for their goals in life. Moreover, the imagery can serve as a way to rehearse new kinds of behavior in a safe environment. This phase also touches on identity and existential meaning, and the chosen music stimulates the patient to reflect upon on these cornerstones of life. The therapy course is brought to a closure, and the focus is on how the patient can go on with their life.

The described protocol is adapted to the specific needs of the patients. In case the patient cannot tolerate music at all as part of being hypersensitive to sound (which can happen due to insomnia, pain or re-traumatization), there can be sessions with only verbalization and guided imagery without music. In case the music therapist suggests that live music would be a more adequate choice than recorded music in terms of attunement to the patient, or should the patient be more able to tolerate live music, the music therapist can use their own voice and musical instruments and create music on the spot to facilitate the inner experience of the patient (as there has been examples of in the pilot study). Accordingly, patients who can tolerate short GIM (longer music and verbal interaction during the music) are offered this option.

### Standard psychological treatment

Standard psychological treatment in the Clinic for Traumatized Refugees is inspired from a broad range of theoretical models such as narrative therapy, cognitive therapy, social psychology and neuro-affective therapy. EMDR is part of the treatment options in the clinic, but is *not* offered to participants in the trial.

A therapy course with verbal therapy is based on a phased treatment of traumatic experiences. The therapy course is adapted to the individual needs of the patient and their symptom load. The overall goals are alleviation of symptoms and normalization; and aiding the patient to understand that symptoms are normal reactions to abnormal incidents. Another goal is to promote patients’ reflection, making way for new insights and decrease conditional reflexes. Phase 1 is focused on general stabilization and basic resourcing and the buildup of trust, making way for the formation of a therapeutic alliance. The work is directed to strengthening the daily level of functioning, to learn techniques to regulate affects, increase affect tolerance, and to create a common understanding of symptoms and discomforts. In phase 2 a processing of traumatic memory is taking place, enabling the patient to break with avoidance behavior, and begin integrating the traumatic memories in the life narrative. Phase 3 includes personal integration and rehabilitation. The phases are not necessarily carried out in a sequence, where one phase comes to an end before the next begins, but rather the phases tend to overlap each other throughout the course of therapy.

If a patient wishes to stop treatment, the clinical team find another suitable treatment modality.

### Assessment of treatment fidelity

Both music therapists and psychologists receive clinical supervision from experienced supervisors. All clinicians report on each session in a special field in the data collection system directly after the session. In this way treatment fidelity can be monitored throughout the study, and violations can be reported. Additionally, the music therapists complete notes of music pieces and inductions accompanying the music listening, and the absence of music listening in a treatment can be noticed.

## Outcome measures

The primary outcome measure is the therapist-administered Harvard Trauma Questionnaire (HTQ) [71], demonstrating an acceptable reliability in different languages, including Arabic [72, 73]. The first 16 items of part IV are used, describing to which degree the participants felt disturbed by trauma symptoms corresponding with the PTSD diagnosis in DSM-IV. The scale has three subscales: avoidance, hypervigilance and intrusion.

Eight of the HTQ questions are included in the scale PTSD-8 [74], which is administered at the beginning of sessions 5 and 10 (of 16 sessions) to monitor the effect on trauma symptoms during treatment.

### Secondary outcomes

The secondary outcomes assess changes in attachment and dissociation, both factors have been associated with PTSD and Complex PTSD, playing a role for the therapeutic alliance, relational capacities and ongoing development of integrative capacity in the patient. The Revised Adult Attachment Scale (RAAS) [75] is a revision of the original Adult Attachment Scale evaluating the experience of emotional closeness or distance with 18 questions which are answered on a Likert scale with 5 points ranging from “right for me” to “not at all right for me.” The questionnaire indicates whether a person has a predominantly safe, defensive/anxious or dependent attachment style. Safe attachment measured with RAAS correlates negatively with the PTSD diagnosis [76].

Two dissociation scales are included in the study. The Dissociative Symptoms Scale (DSS) [77] evaluates moderate to severe levels of depersonalization, de-realization, gaps in awareness or memory, and dissociative re-experiencing. The DSS is applied with the permission from the developers. The 20 questions are answered in relation to the amount of time that the person experiences each symptom on a 5-point Likert scale ranging from “not at all,” “once or twice a day” to “more than once a day.” The scale shows good psychometric properties [77].

The Somatoform Dissociation Questionnaire (SDQ-20) [78, 79] is a supplementary scale for the evaluation of somatic dissociation, but going a little more in depth. The 20 questions ask about dissociative symptoms that are evaluated on a 5-point Likert scale, ranging from “does not at all fit with me” to “fits extremely well with me.” If an item is acknowledged as fitting for the person, there is an additional question to whether a physician has provided a physical diagnosis that explains the symptom or not. The scale has good psychometric properties and has been found to correlate with self-reported traumatization [78, 80].

The WHO Well-being-5 (WHO-5) is a short form that allows information of general health and absence of distress to appear [81]. The scale consists of five questions measuring quality of life and well-being (joy, energy, healthy rest, motivation and meaningful activities).

At the end of each session a session evaluation is carried out. Music therapy patients are asked to what degree they have used the music method since their last session. All patients are asked to rate to what extent they feel understood and heard by the therapist (0–10) and how helpful they find the session (0–10) to be. They are also asked to name the most important themes of the session, and what they think they will remember/tell their spouse when returning home. The music therapists are collecting data about the use of music, intervention and themes of the session.

### Translation of scales

All scales are available in Danish, English and Arabic. The author SM performed a translation of SDQ-20, DSS-20 and RAAS into Arabic together with an expert group of experienced Arabic-Danish translators, and the author BB performed a translation of SDQ-20 from Swedish to Danish with the help of a Swedish health-informed translator living in Denmark. The translations were back-translated and checked for misspellings and misinterpretations following the guidelines for translation of research questionnaires (Process of translation and adaptation of instruments, WHO, n.d.).

### Explorative outcome measure: assessment of levels of neuropeptides

As mentioned in the background section several studies have assessed oxytocin in connection with PTSD patients, but only after a single intervention. In the present study, we chose to assess the change in in neuropeptide concentrations following a single intervention and the possible changes in basic levels of the neuropeptides oxytocin, beta-endorphin and substance P after treatment. This remains an exploratory part of the trial.

The collection of saliva is ethically less invasive than blood sample collection, it is self-administered and it takes less than a minute to collect a sample.

### Collection and analysis of saliva samples

The collection of saliva samples is carried out by the therapists in the project. A description of the collection procedure is available for all therapists. 1–2 ml of saliva are collected from the patients in a tube (Disposable plastic tube, Thermo Scientific Nunc 345,608, 14 ml) or a small petri bowl (Thermo Scientific Nunc IVF ICSI Dish). The patients are given the possibility to be alone in the room while collecting saliva. The therapist fills out a label with patient ID, time and date and data time point. The female patients are asked whether they are menstruating, and this is noted on the label, as it could influence the hormone levels. The samples are stored immediately in a freezer at − 18–20 °C. The samples are transported to the Translational Unit, Neuropsychiatry Unit (TNU), Aarhus University in a flamingo box with cool freeze bricks (Farusa emballage, foam refrigerant bricks) at a temperature of − 70 °C. At TNU they are stored at − 80 °C until further handling. When all samples have been collected, the levels of oxytocin, beta-endorphin and substance P will be analyzed using a Luminex and a Milliplex kit (Human Neuropeptide Magnetic Bead Panel; Neuroscience Multiplex Assay (HNPMAG-35 K)).

#### Procedures

1. After assessment by the visitation team and at the team conference, eligible patients are invited to participate in the study by one of the three music therapists/researchers
2. The patient is informed orally and in writing about the study
3. If the patient accepts participation, informed consent is signed by patient and therapist. If participation is rejected the patient is offered other treatment in the clinic
4. Baseline measurement is carried out by the music therapists/researchers. Information about health according to height, weight, exercise, use of alcohol, smoking habits, symptoms and medication is collected during the visitation procedures in the clinic, and transferred to the dataset by the researchers. All data collection is carried out on laptops with a data collection environment called Xpsy (see below in the “Data management” section). Demographic data and information relevant for the trauma history are collected and scored regarding age, gender, country of origin, native language, education, civil state, number of children at home, whether the patient has been sexually or physically abused during their childhood, imprisonment (number of weeks), exposure to torture, number of weeks on flight, number of weeks in refugee camps and/or asylum centers. The patient fills out self-report questionnaires and a saliva sample is collected. The scoring of the primary questionnaire HTQ is done by a psychologist or trained music therapist, as it requires specialist knowledge and training, and is based on an interview with the patient. The secondary questionnaires are filled out in the presence of one of the researchers or with the presence of a translator trained in assisting the scoring of the questionnaires
5. The patient is randomized to treatment with music therapy or standard treatment
6. Treatment is carried out according to the descriptions under “Interventions”
7. Patient data are recorded in all sessions (session evaluation), session data regarding use of music and themes for the therapy are collected by the music therapists with the help of translators
8. Data collection (PTSD-8) is carried out in sessions 6 and 11 and saliva samples are collected in sessions 3 and 14 (see “Outcome measures” and the flow chart in Fig. 1)
9. After the last session, a post-treatment data collection session is scheduled, where all questionnaires are filled out, HTQ is scored by an external psychologist (and translator) who is not a part of the treatment team and who is blinded to the patient’s treatment group. Three questions regarding the patient’s own evaluation of their current life situation are posed by the music therapist/researcher. In order to leave out confounders of diurnal variation, the time of the meeting is scheduled so that the collection of saliva can occur at the same hour as the baseline sample was collected. Any need for additional treatment is assessed by the clinical team. If additional psychotherapeutic treatment is needed, the participant is excluded from follow-up measurement. All participants who have completed the protocol are invited to a 6-month follow-up session, where questionnaires and saliva samples are collected, with an external psychologist assisting in scoring HTQ, and a trained translator, who is blinded to the affiliation, assisting in scoring the remaining questionnaires

Adherence to treatment is monitored through the evaluation of sessions, and in case of dropout participants are asked about their reasons for stopping treatment, if possible. A research log including dropout information is kept by the researcher team. Additional sessions in the clinic during participation (such as body therapy or social counseling) and change of medication are followed in the patient journal and scored in the data collection environment.

### Data management

All data related to the study are stored with highest possible level of security. Questionnaire data, session evaluation and demographic data (including health and trauma history data) are typed into a database with the program Xpsy, which is a quality assurance system for psychiatric clinics developed by PsyMeta Gmbh by Franz Fischer, Shafisheim in Switzerland (https://www.xpsy.eu/). It is administered by the co-researcher and data manager (second author SM). All the questionnaires are set up in electronic versions in the program in three languages, and are stored in the database as soon as they are typed in. The program ensures that all data can be typed by the participants and/or translators without missing any questions, the data time point for the single participant is clearly indicated and the dates of entering the system can be monitored. All patients have their own login based on ID number. Researchers have a common code to access the participant’s actual session or questionnaire session, and typing in of demographic data.

### Data confidentiality

The project is approved by the Danish data management authorities “Datatilsynet” under the protocol number REV-50-2014. Data are stored until the completion of analysis and are then deleted. Saliva samples are stored until 2027 in case of the need to go back and do additional analyses. In that case all participants will be asked for additional consent.

The research data typed into the Xpsy database are stored on a secured server that is placed in a locked cabinet. Confidential data regarding patients, such as list of patients in the study, reasons for decline of participation for eligible patients and list of completed saliva samples are stored at a protected website for clinicians at the Clinic of Traumatized Refugees. Any other data, such as informed consent contracts, clinical notes, patients’ drawings, are stored in locked cabinets. Patient data related to treatment, other than research data, are stored in the patient database OPUS, Region Zealand, or after 25. November 2017 in the application “Sundhedsplatformen.”

Saliva samples are stored in research freezers placed in locked local facilities.

For the data analysis, the members of the Steering Group and the group of three music therapist/researchers will have access to data, stored under ID numbers in the Xpsy environment. Access will also be granted to the statistical consultant who works in the same organization (Region Zealand). A signed data agreement contract is made between the Regional Zealand and Aarhus University for exchanging information on the saliva data.

### Translators

Arabic translators are included for Arabic-speaking participants as needed. The translator is physically present during translation. The translators are trained in the management of questionnaires and the Xpsy environment for data collection. Translators assist the completion of questionnaires for Arabic-speaking participants. All translators are asked about their educational background and experience of translation, so that only translators with adequate education and experience with psychotherapy are used. All translators used in music therapy treatment are instructed in translating during induction and music listening, and they receive a self-experience of music and imagery to educate them in the special use of the voice during music and imagery with participants.

#### Statistical methods

Statistical analysis will be carried out in the statistical environment R [82] in cooperation with the statistical department in the research unit of Region Zealand and PFI Region Zealand (Production, Research and Innovation Unit). Data will be treated according to the intention-to-treat principle. Analysis of all data will take place after the conclusion of data collection. Following the initial screening of the data significance tests concerning differences between standard treatment and music therapy will be carried out in order to assess the non-inferiority of music therapy. Significance and variance for the primary outcome measure will be calculated with analysis of covariance (ANCOVA), including data from five measuring points. Secondary questionnaire outcome data will also be calculated with ANCOVA, using three data points. Correlations between trauma symptoms, attachment, dissociation and demographic parameters are investigated. A regression analysis will be applied to look for predictors of improvement of trauma symptoms (HTQ) and change of attachment style, as well as predictors for improvement connected to treatment (music therapy or standard treatment). Analysis of hormones will be split up in an ANCOVA testing variance between groups and with time (baseline to follow-up), and simple significance tests of change after single sessions (between the third and 14th session and between the interventions). Session satisfaction data will be treated with descriptive statistical methods.

#### Power calculation

In order to estimate the level of power we reviewed randomized and non-randomized trials where refugees suffering from PTSD were treated with stabilization and trauma exposure strategies with cognitive and narrative elements corresponding to the standard treatment in our study, and where the Harvard Trauma Questionnaire was used to measure changes in trauma symptoms (the primary outcome in the current study). The variations in the studies were considerable, and we chose to only look at studies that had a number of sessions that were similar to the current study, and where the mean baseline value of HTQ were around 3.3, a value that we found to correspond to our population in the pilot study [18, 62, 83–86]. Non-significant differences between 0.1 and 0.5 was found in HTQ from pre to post treatment. Based on our clinical experience and data from these studies, we estimate a clinical insignificant difference of 0.3 as the maximal difference between music therapy and standard treatment to confirm the non-inferiority hypothesis. A mean standard deviation of 0.48 on post-scores of HTQ was calculated from the studies referred above.

The power calculation was based on a significance level of 0.05, power 0.08, *d* = 0.3 and *SD* = 0.48. The calculation was carried out with software from Epi-info 7 (http://wwwn.cdc.gov/epiinfo/) in cooperation with Department for Statistics, Psychiatric Research Unit, Region Zealand and PFI.

The result indicates a minimum of 64 participants (32 in each group).

Adherence to music therapy has been found to be good in psychiatric patients with a low dropout rate (11.5%) [87]. In three former randomized clinical studies on psychological treatment of refugees with large samples low dropout rates (7–10%) were demonstrated at follow-up [21, 83, 88].

We have, therefore, chosen to include a dropout rate of 10% and thus end up with *n* = 70 (35 in each group).

#### Randomization

Randomization is carried out with the help of the randomization software Sealed Envelope (https://sealedenvelope.com/). Stratification is applied regarding geography (three different locations) and gender (male/female). Within the strata, random permuted blocks of even length (blocks of four or six participants) are used. When a participant has given informed consent and completed baseline measures with one of the three music therapists who takes care of the research procedures (one in each location), the music therapist/researcher logs on to the randomization website and types patient ID number, gender and location. Information of the treatment group is provided immediately on the website and is also sent by email to the researcher. The status of the patient is typed into the Xpsy database, and the patient is referred to either music therapy or standard treatment at the location.

#### Blinding

There is no blinding connected to the randomization of intervention. Questionnaire data are blinded to all clinicians who are performing the treatment and data collection in the project, so that none of the clinicians or researchers have access to completed questionnaire data from their own patients or any other participant in the trial. This ensures that the clinical processes are not influenced by the outcome results data.

External psychologists are called in to assist the scoring of HTQ post treatment and follow-up, they are blinded to the treatment of the participant. Data remain concealed until the entire trial is completed.

#### Potential harms

Potential harms of the trial can occur as harms of the interventions as well as harms of the research procedures. Music therapy is a relatively new treatment modality for refugees, and the art of choosing music for the right phase of treatment is still being developed. According to the pilot study, the treatment method is not harmful when used with care and ongoing attunement to the needs of the patient, but the music therapists must pay attention to avoid adverse reactions in case of hypersensitivity to sound, restimulation of trauma by using too loud or dynamic pieces of music, or restimulation of trauma in former musicians or persons who have been tortured with sound or music. Music listening that triggers trauma memory has been found to happen frequently and, therefore, the music therapists have to be specifically trained when working with this clinical group. Trauma exposure with music is very effective, but it requires that both the patient and the therapist can work together to keep arousal at a manageable level. The patient is given control over music choice and volume, and is educated to give feedback before, during and after music listening, as well as how to use music safely at home.

It is well-known, that the exposure phase of trauma treatment both in music therapy and standard treatment will stir up traumatic memories which can worsen the symptoms for a period. The patients are informed about this and supported to cope with the symptoms. The therapists can go back to stabilization work whenever needed to facilitate a safe therapeutic course of treatment. Some of the patients express a need for longer treatment periods, that collides with the six months’ follow-up period without treatment. The therapists normally have the same amount of time for each patient as planned for in the study, and the closure of therapy is planned for with care. However, some patients happen to be re-traumatized by external events, and in such cases the treatment team of the clinic can estimate whether they can receive additional treatment and be excluded from follow-up measures in the study.

Regarding the potential harms of working with the research questionnaires, the trauma symptoms questionnaire and the dissociation scales sometimes can be challenging for the patients as they are reminded about traumatic incidents. The number of self-report questionnaires utilized has been kept at a reasonable number, but in case the patients experience fatigue or confusion, breaks are introduced, or the scoring is extended to two different days. Even though the sampling of saliva is non-invasive and quick, some patients experience nausea or disgust, or they are reminded of traumatic experiences. Patients who are not able to give saliva are respected, and the procedure is cancelled. Any negative reaction is processed by the therapist/researcher.

The plan for monitoring and acting on any incidents of harm or unintended reactions is embedded in the clinical emergency report system. The clinicians monitor adverse patient reactions, report them in the journal system, and also immediately report to the leading physician, who has clinical responsibility. Patients can telephone the clinic at any time during opening hours to receive support and have additional appointments. Incidents will also be discussed on the weekly clinical team meeting and by the team of music therapist researchers at monthly meetings.

#### Auditing

All clinicians of the trial (psychologists and music therapists) have meetings at the beginning of the trial to be informed about procedures and to resolve questions and problems related to the trial conduct. The group of music therapist researchers meets once a month to coordinate and monitor the trial. The Steering Committee of the study meets every four months to oversee the development of the study. Both groups include investigators as well as clinicians, but the researchers have no access to data before the end of data collection.

#### Protocol amendment

The protocol cannot be changed without corresponding with the Regional Scientific Committee. Any changes to the protocol have to be approved by the Regional Scientific Committee, following the regulations for protocol amendment applications. Protocol amendment is also reported to ClinicalTrials.com.

#### Dissemination

Both positive and negative results of the trial will be reported in the relevant scientific journals and at international conferences. A summary of the results will also be published in the healthcare system and to the public. A conference day for refugee clinics in the country and neighboring countries is planned for. A poster with a summary of the results will be placed in the refugee clinics and translated into Arabic.

## Discussion

### Music therapy and mechanisms of change

Brain research on the perception of music indicates that music positively affects brain chemistry associated with stress, immune defense, reward and attachment systems [29], and that music strongly affects and changes activity in brain areas connected to emotion regulation and social response such as the limbic and paralimbic structures [89]. PTSD is connected to stress-related loss of hippocampal mass [90] and hypervigilance related to an increased amygdala-hippocampus connectivity [91]. Brain studies have shown how music can enhance the connection between prefrontal areas and amygdala/hippocampus and thereby calm down hypervigilance and enhance reflectivity and cognitive processing of emotions [92]. Furthermore, music listening has been shown to reduce stress and enhance emotional responses, such as joy, peacefulness and calmness [93]. A recent functional magnetic resonance imaging (fMRI) study compared guided imagery, music alone, GIM and a control group in participants recalling personal episodic memory with negative-emotion. The study indicated that GIM was most effective in the processing of traumatic memories affecting cortical and subcortical structures and functions [94]. Music can intervene in the avoidance response seen in many PTSD patients: “Superficial amygdala, nucleus accumbens and mediodorsal thalamus constitute a network that modulates approach-withdrawal behavior in response to socio-affective cues such as music.” [93].

In order to understand possible mechanisms of music therapy in the treatment of refugees with PTSD, the theory of neuroception [95, 96] might explain how music can decrease hypervigilance. Trauma disrupts basic autonomic regulation, where exaggerated sympathetic responses known as fight and flight, and parasympathetic responses known as freeze and feign death/total submission occur. Based on studies of heart rate variability, Porges argued that the mammal parasympathetic branch is divided into a dorsal branch associated with immobility responses and a ventral part associated with “social engagement.” He showed how facial muscles, ears, eyes, heart and stomach functions are connected, and that face-to-face interaction and communication can calm the nervous system down and act as a brake on the heart rate. During stress the ears accomodate for very high and very deep sound frequencies and during deactivation of stress the middle frequency area, such as the human speaking voice, is augmented in the auditory system [97]. Hence, calm music and speaking combined with a thorough attunement to the patient might activate the social engagement system, lead to down-regulation of arousal, and enable the patient to unfreeze and experience aliveness and energy.

The use of music in trauma treatment serves as a way to build up inner resources in the patient necessary for working through the trauma story, such as positive memories, a feeling of strength, a safe place, or the aesthetic experience of music. Exposure is part of many trauma therapies and includes the narration of the trauma story, the re-imagination being part of this retelling. When the narration of trauma episodes is accompanied by music, the music serves both as a holding and structuring framework that keeps the patient from fragmenting. It also helps the stimulation of imagery so that the recalling of trauma memory can change from being stuck in repetitive flashback. With the music as a support, processing of trauma fragments can take place at an implicit level of body sensation and imagery formation, a symbolization process where the memory is transformed into a metaphor [98, 99]. The ability to symbolize an experience allows it to be installed as memory that can be placed in the past instead of occurring as a recurrent flashback experienced as real time. When working with imagination to music, it also seems that the memory of a traumatic episode sometimes begins to transform and the patient imagines a new solution; for example, of escape, control or victory, that allows for the completion of fight and flight actions that were impossible to carry out at the time of the trauma, which, according to Peter Levine, is at the core of the trauma-healing process [100, 101].

### Risk of bias

In a psychotherapy trial such as this, it is often not possible to blind the intervention of the participants, and there can be an influence of their knowledge of being in the intervention or the control group. However, in this trial we investigate two types of treatment in a single clinic with equal dose (16 sessions) and equal weight as primary trauma treatment modalities. According to concealment of allocation, the randomization procedure is generated by computer software at Sealed Envelope, and none of the researchers have any influence over the procedure. Baseline tests are carried out before randomization, so that the allocation does not influence the measurement. Psychologists carrying out data collection concerning the primary outcome measure at post and follow-up times are concealed to the allocation (and the patients are not asked about it). Imbalances between treatment groups are prevented by the use of stratification (gender and location).

As music therapists carry out the information meetings and measurement, the participants might be more motivated for music therapy than standard treatment (comparator), and be more likely to drop out from standard treatment. This could possibly influence the results in favor of music therapy. Adherence to the treatment is secured, as none of the participants can cross over to the other group. If a patient drops out from music therapy and is offered standard treatment, they are excluded from follow-up. If participants are offered other types of treatment in the clinic parallel to the assigned treatment, this will be monitored and an analysis of any group differences will be carried out.

Protocol fidelity is assured by data collection of the therapist notes for all sessions, by team supervision and frequent meetings between music therapists and psychologists and in the group of music therapy researchers. The use of Xpsy for data collection ensures that no participant data are analyzed in the wrong group.

### Limitations and complexities

A number of factors adds to the complexity of the trial and possibly influence the outcome in different ways. According to the treatment recommendations for complex PTSD mentioned in the academic literature review, adequate length of treatment is in the range from 1 to 2 years, compared to our timeframe of 4 to 6 months. Very few of in the target population have simple forms of PTSD, where short-term standard treatments have been proven effective. As studies have shown, complex forms of PTSD and compromised attachment are prevalent in the population. However, the trial is conducted within the premises of Psychiatry in Region Zealand, where the countrywide recommendations are followed. Those recommendations are currently updated. This means that we cannot expect large effect sizes.

Ongoing stressors in participants’ worlds include news and video footage of current bombings of people in their home town or where their friends or family live, confusion about the explicit and implicit rules and norms in their host society, exhaustion from having to follow language training and workplace practice with ongoing PTSD symptoms as well as raising children on a minimal budget, and last but not least the alienation of witnessing a hostile tone towards them from governments as well as citizens in the public news. Such elements make it very difficult to construct a social and psychological space suitable for healing past trauma. Overall, this contributes to a lesser effect size, but the load is spread unevenly and the aim should be to record the most important of these circumstances in each case.

Translators are used with participants, who find it beneficial. In a translated session, the verbal information passed is little less than half of that of a session without, as everything has to be said twice and quite often there is necessary conversation about the meaning of a single sentence. Consequently, the doses of therapy for participants with translators are not comparable to those without translators. There are often limits to the conversation with the translator, who might be a young and relatively inexperienced person with a limited vocabulary on matters relating to psychotherapy and trauma in either one of the languages used. Many of the Arabic-speaking participants do not have Arabic as their mother tongue. There is no officially approved education for translators, the clinic is not free to choose the best available translators, but is bound by an exclusivity contract with one vendor. Most of the translators are bi-lingual persons without any formal training as translator or any higher education in either language. The translator brings their own presence into the therapy room and the therapeutic dyad effectively becomes a triad. The official goal of the translator is not to be personally present, but in a psychotherapy session, where the implicit is just as important as the explicit, this becomes impossible, and the best implicitly present translator is the one who can join the atmosphere that develops in the session and not stick to a rigid pretence of not being there. It follows that translation alone is a complex phenomenon that influences outcome more for some clients and not at all for those who do not use translators. Furthermore, it perhaps influences music therapy and standard treatment differently.

The use of the interdisciplinary team during the course of treatment according to individual needs means that some participants receive more treatment than others, especially so for participants needing physiotherapy or body therapy (psychomotorical therapy). This is usually prescribed for patients having specific and disturbing physical symptoms. Also, advice from a social worker helps clarify issues with authorities and other professionals. The amount of extra treatment and counseling is monitored and recorded in each case.

Another limitation is the broadness of standard treatment; as the psychologists in the clinic work with an integrative approach, it is not possible in this study to compare music therapy with a standardized or manualized psychological treatment. As described in the intervention section, the main perspectives adapted in the clinic are a flexible adaptation of CBT to severely traumatized patients from diverse cultures and narrative therapy (which is focused on helping the patient to create a narrative of the life story that helps them to be able to make meaning and live on after trauma; it is by the way not the same as NET). In reviews of psychological treatment with refugees, the two treatments with highest effect sizes are culturally sensitive CBT and NET, but they have been conducted almost exclusively by the same two groups of researchers, and have been criticized for having low-quality evidence [12]. Studies of other types of intervention, including multimodal therapy, reach an average medium-large effect size, and this is also what we will expect from the outcomes of the present study. A recent review by Tribe et al. concluded that refugee research should include more “real-world” multidisciplinary interventions that better model clinical practice, which we agree upon from our clinical experience [102].

Several of the outcome measures were not used in our pilot study, so we do not have previous experience with the use of the questionnaires in the present context. Furthermore, the translations of the scales that we performed for the study has not been validated. According to the physiological outcome measurement, one limitations is that the existing knowledge is limited, and no reference levels are established, making the results provisional and exploratory. To our knowledge no previous studies of substance P in relation to treatment have been carried out.

### Implications

We expect that the trial will demonstrate that music therapy can be a feasible and effective intervention for the treatment of refugees, and that we will know more about which subgroups of patients will have special benefit of music therapy. We also hope that the trial will provide arguments for extended use of music therapists in the treatment of refugees with severe trauma. If correlations between physiological and self-report measures can be found this will support the strength of the trial, and make way for new knowledge about trauma and biomarkers. As the intervention group is asked to use music at home as a tool for affect and arousal regulation, increased knowledge about music as a health resource for traumatized refugees will be provided. The study on the use of music therapy hopefully will add new possibilities for the treatment of this vulnerable population, and thereby be helpful for the increase of refugee health and integration in the society.

### Trial status

The current protocol version has number 04, and is dated 30 March 2016. Recruitment began on 9 May 2016. We estimate that recruitment will be completed by 1 March 2018.

### Additional files

Additional file 1: Standard Protocol Items: Recommendations for Interventional Trials (SPIRIT) 2013 Checklist: recommended items to address in a clinical trial protocol and related documents*. (DOCX 45 kb)

Additional file 2: Informed consent (English version). (DOCX 167 kb)

## Appendix 1

Locations of the three units of the clinic:

1. Clinic for Traumatized Refugees, Glæisersvej 50, 4600 Køge
2. Clinic for Traumatized Refugees, Fælledvej 6, 1., 4200 Slagelse
3. Clinic for Traumatized Refugees, Færgegaardsvej 15, 4760 Vordingborg

## Appendix 2

Music on the CD for home listening/assessment

1. Satie, E. (1990). *Trois Gymnopédies nr. 1* (Klara Körmendi, piano). On: *Piano Works (Selection)* (CD), Label: Naxos

2. Enya (1988). *Watermark*. On: *Watermark* (CD). Label: WEA

3. Pärt, A. (1994) *Spiegel im Spiegel* (Tasmin Little, violin; Martin Roscoe, piano). On: *Fratres* (CD). Label: EMI Classics

4. Richter, M. (2015). *Dream 13* (minus even). On: *Sleep*, (CD). Label: Deutsche Grammofon

5. Deva Premal (2002). *Om Namoh Bhagavate*. On: *Embrace* (CD). Label: White Swan

6. Norge, K. (2013*). Homage to Life* (opus 11, No.1). On: *Fiesta* (CD). Label: Digidi

7. Tekbilek, OF. (1994). *Moment of Doubt*. On: *Whirling* (CD). Label: Celestial Harmonies

## Abbreviations

ANCOVA: Analysis of covariance
CBT: Cognitive behavioral therapy
C-PTSD: Complex post-traumatic stress disorder
DSM-IV: *Diagnostic and Statistic manual of Mental Disorders, version 4*
DSS: Dissociation Symptoms Scale
EMDR: Eye movement desensitization and reprocessing
HTQ: Harvard Trauma Questionnaire
ICD-10: *International Classification of Diseases*
NET: Narrative exposure therapy
PTSD: Post-traumatic stress disorder
RAAS: Revised Adult Attachment Scale
SDQ: Somatoform Dissociation Questionnaire
TMI: Trauma-focused Music and Imagery
WHO: World Health Organization
