# Supplementary material for: Music therapy versus treatment as usual for refugees diagnosed with posttraumatic stress disorder (PTSD): study protocol for a randomized controlled trial
Source: Trials. 2018 May 30;19:301. doi: 10.1186/s13063-018-2662-z (PMC5977477; doi:10.1186/s13063-018-2662-z)
Supplement: Supplementary file 1 — Standard Protocol Items: Recommendations for Interventional Trials (SPIRIT) 2013 Checklist: recommended items to address in a clinical trial protocol and related documents*. (DOCX 45 kb) [file 13063_2018_2662_MOESM1_ESM.docx]

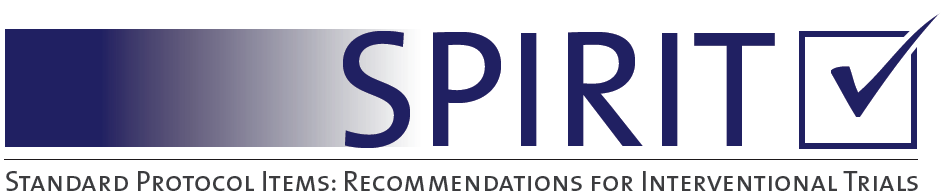


SPIRIT 2013 Checklist: Recommended items to address in a clinical trial protocol and related documents*

# Section/item Item No

**Description Addressed on**

**page number**

**Administrative information**

| Title | 1 | Descriptive title identifying the study design, population, interventions, and, if applicable, trial acronym | ____1________ |
| --- | --- | --- | --- |
| 1 | 2a | Trial identifier and registry name. If not yet registered, name of intended registry | ___1-2________ |
|  | 2b | All items from the World Health Organization Trial Registration Data Set | _____________ |
| Protocol version | 3 | Date and version identifier | ___32_________ |
| Funding | 4 | Sources and types of financial, material, and other support | __31__________ |
| Roles and | 5a | Names, affiliations, and roles of protocol contributors | _33-34________ |

responsibilities

5b Name and contact information for the trial sponsor ____31_________ 5c Role of study sponsor and funders, if any, in study design; collection, management, analysis, and

interpretation of data; writing of the report; and the decision to submit the report for publication, including whether they will have ultimate authority over any of these activities

5d Composition, roles, and responsibilities of the coordinating centre, steering committee, endpoint adjudication committee, data management team, and other individuals or groups overseeing the trial, if applicable (see Item 21a for data monitoring committee)

____31_________

____33_______

| **Introduction** |  |  |  |
| --- | --- | --- | --- |
| Background and rationale | 6a | Description of research question and justification for undertaking the trial, including summary of relevant studies (published and unpublished) examining benefits and harms for each intervention | __2-9___________ |
|  | 6b | Explanation for choice of comparators | ____8_________ |
| Objectives | 7 | Specific objectives or hypotheses | ___9__________ |
| Trial design | 8 | Description of trial design including type of trial (eg, parallel group, crossover, factorial, single group), allocation ratio, and framework (eg, superiority, equivalence, noninferiority, exploratory) | ___9-10_________ |

# Methods: Participants, interventions, and outcomes

Study setting 9 Description of study settings (eg, community clinic, academic hospital) and list of countries where data will be collected. Reference to where list of study sites can be obtained

Eligibility criteria 10 Inclusion and exclusion criteria for participants. If applicable, eligibility criteria for study centres and individuals who will perform the interventions (eg, surgeons, psychotherapists)

Interventions 11a Interventions for each group with sufficient detail to allow replication, including how and when they will be administered

11b Criteria for discontinuing or modifying allocated interventions for a given trial participant (eg, drug dose change in response to harms, participant request, or improving/worsening disease)

11c Strategies to improve adherence to intervention protocols, and any procedures for monitoring adherence (eg, drug tablet return, laboratory tests)

___10-11_____

____11, 14_______

____11-16_______

___16, 25________

___-__________

11d Relevant concomitant care and interventions that are permitted or prohibited during the trial ___13__________

Outcomes 12 Primary, secondary, and other outcomes, including the specific measurement variable (eg, systolic blood

pressure), analysis metric (eg, change from baseline, final value, time to event), method of aggregation (eg, median, proportion), and time point for each outcome. Explanation of the clinical relevance of chosen efficacy and harm outcomes is strongly recommended

Participant timeline 13 Time schedule of enrolment, interventions (including any run-ins and washouts), assessments, and visits for participants. A schematic diagram is highly recommended (see Figure)

___18-20_______

_____10, 20-21__

Sample size 14 Estimated number of participants needed to achieve study objectives and how it was determined, including clinical and statistical assumptions supporting any sample size calculations

_____23-24_____

Recruitment 15 Strategies for achieving adequate participant enrolment to reach target sample size ____12_________

# Methods: Assignment of interventions (for controlled trials)

Allocation:

Sequence generation

Allocation concealment mechanism

16a Method of generating the allocation sequence (eg, computer-generated random numbers), and list of any factors for stratification. To reduce predictability of a random sequence, details of any planned restriction (eg, blocking) should be provided in a separate document that is unavailable to those who enrol participants or assign interventions

16b Mechanism of implementing the allocation sequence (eg, central telephone; sequentially numbered, opaque, sealed envelopes), describing any steps to conceal the sequence until interventions are assigned

____24________

____24________

Implementation 16c Who will generate the allocation sequence, who will enrol participants, and who will assign participants to interventions

Blinding (masking) 17a Who will be blinded after assignment to interventions (eg, trial participants, care providers, outcome assessors, data analysts), and how

17b If blinded, circumstances under which unblinding is permissible, and procedure for revealing a participant’s allocated intervention during the trial

___24____

___24__________

___-__________

# Methods: Data collection, management, and analysis

Data collection methods

18a Plans for assessment and collection of outcome, baseline, and other trial data, including any related processes to promote data quality (eg, duplicate measurements, training of assessors) and a description of study instruments (eg, questionnaires, laboratory tests) along with their reliability and validity, if known.

Reference to where data collection forms can be found, if not in the protocol

18b Plans to promote participant retention and complete follow-up, including list of any outcome data to be collected for participants who discontinue or deviate from intervention protocols

____20-22_______

___21__________

Data management 19 Plans for data entry, coding, security, and storage, including any related processes to promote data quality (eg, double data entry; range checks for data values). Reference to where details of data management procedures can be found, if not in the protocol

Statistical methods 20a Statistical methods for analysing primary and secondary outcomes. Reference to where other details of the statistical analysis plan can be found, if not in the protocol

____21-22_______

____23_________

20b Methods for any additional analyses (eg, subgroup and adjusted analyses) _____23________

20c Definition of analysis population relating to protocol non-adherence (eg, as randomised analysis), and any

statistical methods to handle missing data (eg, multiple imputation) ____-_________

# Methods: Monitoring

Data monitoring 21a Composition of data monitoring committee (DMC); summary of its role and reporting structure; statement of whether it is independent from the sponsor and competing interests; and reference to where further details about its charter can be found, if not in the protocol. Alternatively, an explanation of why a DMC is not needed

21b Description of any interim analyses and stopping guidelines, including who will have access to these interim results and make the final decision to terminate the trial

Harms 22 Plans for collecting, assessing, reporting, and managing solicited and spontaneously reported adverse events and other unintended effects of trial interventions or trial conduct

Auditing 23 Frequency and procedures for auditing trial conduct, if any, and whether the process will be independent from investigators and the sponsor

____22_________

____33_________

___26__________

___26__________

# Ethics and dissemination

Research ethics approval

1. Plans for seeking research ethics committee/institutional review board (REC/IRB) approval ____32_________

Protocol amendments

1. Plans for communicating important protocol modifications (eg, changes to eligibility criteria, outcomes, analyses) to relevant parties (eg, investigators, REC/IRBs, trial participants, trial registries, journals, regulators)

_____26________

Consent or assent 26a Who will obtain informed consent or assent from potential trial participants or authorised surrogates, and how (see Item 32)

26b Additional consent provisions for collection and use of participant data and biological specimens in ancillary studies, if applicable

Confidentiality 27 How personal information about potential and enrolled participants will be collected, shared, and maintained in order to protect confidentiality before, during, and after the trial

____12_________

_____22________

____22_________

Declaration of interests

28 Financial and other competing interests for principal investigators for the overall trial and each study site ____32_________

Access to data 29 Statement of who will have access to the final trial dataset, and disclosure of contractual agreements that limit such access for investigators

____22_________

Ancillary and post- trial care

30 Provisions, if any, for ancillary and post-trial care, and for compensation to those who suffer harm from trial participation

____-_________

Dissemination policy 31a Plans for investigators and sponsor to communicate trial results to participants, healthcare professionals, the public, and other relevant groups (eg, via publication, reporting in results databases, or other data sharing arrangements), including any publication restrictions

___26-27________

31b Authorship eligibility guidelines and any intended use of professional writers ____-_________ 31c Plans, if any, for granting public access to the full protocol, participant-level dataset, and statistical code ____-_________

# Appendices

Informed consent materials

1. Model consent form and other related documentation given to participants and authorised surrogates _____43________

Biological specimens

1. Plans for collection, laboratory evaluation, and storage of biological specimens for genetic or molecular analysis in the current trial and for future use in ancillary studies, if applicable

__19-21_________

*It is strongly recommended that this checklist be read in conjunction with the SPIRIT 2013 Explanation & Elaboration for important clarification on the items. Amendments to the protocol should be tracked and dated. The SPIRIT checklist is copyrighted by the SPIRIT Group under the Creative Commons “Attribution-NonCommercial-NoDerivs 3.0 Unported” license.
