# Supplementary figures and images for: Music therapy versus treatment as usual for refugees diagnosed with posttraumatic stress disorder (PTSD): study protocol for a randomized controlled trial
Source: Trials. 2018 May 30;19:301. doi: 10.1186/s13063-018-2662-z (PMC5977477; doi:10.1186/s13063-018-2662-z)

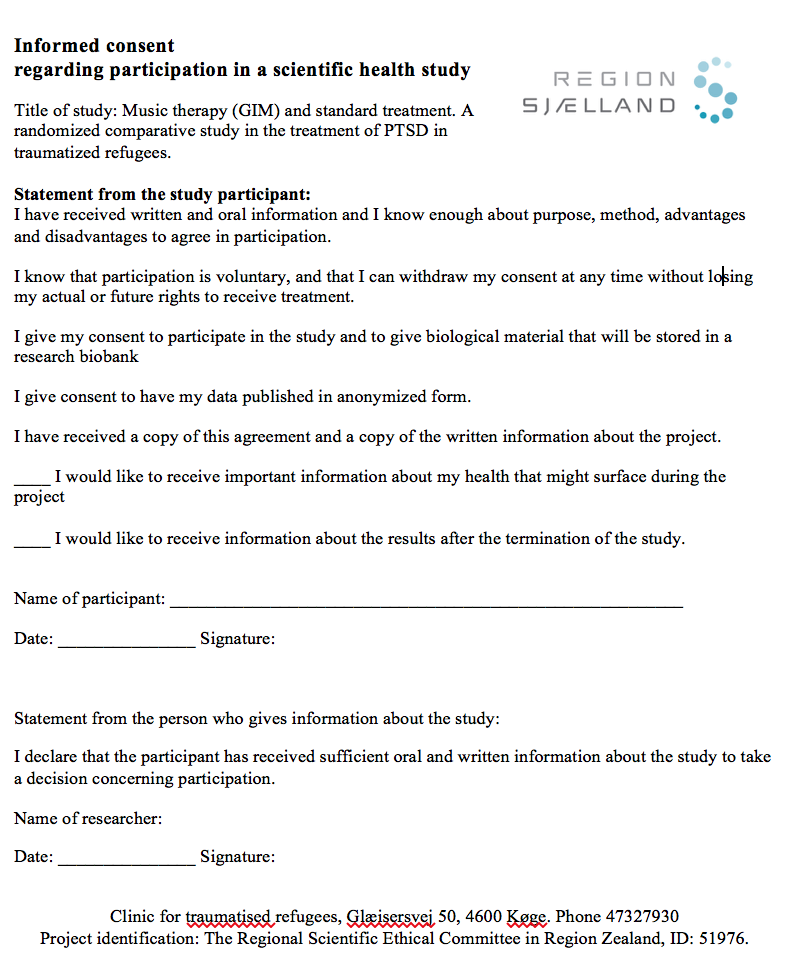

Supplement: Supplementary file 2 — Informed consent (English version). (DOCX 167 kb) [file 13063_2018_2662_MOESM2_ESM.docx]
